# The mechanism and potential therapeutic target of piezo channels in pain

**DOI:** 10.3389/fpain.2024.1452389

**Published:** 2024-09-27

**Authors:** Yi Xu, Yuheng Wang, Shuchong Mei, Jialing Hu, Lidong Wu, Luyang Xu, Lijie Bao, Xiaowei Fang

**Affiliations:** ^1^ Department of Emergency Medicine, Jiangxi Medical College, The Second Affiliated Hospital of Nanchang University, Nanchang University, Nanchang, China; ^2^ Jiangxi Medical College, The Second Affiliated Hospital of Nanchang University, Nanchang University, Nanchang, China

**Keywords:** *Piezo*1, *Piezo*2, therapeutic target, pain, Ca^2+^

## Abstract

Pain is a common symptom of many clinical diseases; it adversely affects patients’ physical and mental health, reduces their quality of life, and heavily burdens patients and society. Pain treatment is one of the most difficult problems today. There is an urgent need to explore the potential factors involved in the pathogenesis of pain to improve its diagnosis and treatment rate. *Piezo*1/2, a newly identified mechanosensitive ion channel opens in response to mechanical stimuli and plays a critical role in regulating pain-related diseases. Inhibition or downregulation of *Piezo*1/2 alleviates disease-induced pain. Therefore, in this study, we comprehensively discussed the biology of this gene, focusing on its potential relevance in pain-related diseases, and explored the pharmacological effects of drugs using this gene for the treatment of pain.

## 1 Introduction

Pain is commonly defined as an unpleasant sensory and emotional experience linked to or similar to, the experience of actual or potential tissue damage (1). Pain is classified based on several factors. The time of onset is one such factor, with pain categorized as acute or chronic. Another factor is the underlying cause of the pain, which can be classified as inflammatory, neuropathic, or cancer-related. Pain can be categorized according to severity, ranging from mild to moderate to severe (2). In this study, we focused on pain resulting from various etiologies. Many diseases are known to cause pain; however, the areas of research that have garnered particular attention are inflammatory, neuropathic, and cancerous pain (3). The pain stemming from these causes is predominantly chronic in nature and may be accompanied by additional symptoms such as dizziness, nausea, vomiting, and anorexia (4). Undoubtedly, the experience of pain creates significant obstacles to daily activities (5), however, for a prolonged period, there was a lack of distinct diagnostic criteria for pain. Until the release of the International Classification of Diseases (ICD) in 2018, pain was not systematically classified and the diagnostic criteria for each type of pain were not summarized. This classification system provides a standardized framework for the diagnosis and treatment of various types of pain (6).

*Piezo*1/2 has been identified as a crucial element in cellular activity, and any mutations in the *piezo*1/2 gene can cause diseases in different parts of the human body. One illustrative example of this class of disorders is Hereditary Xerocytosis (HX), a condition precipitated by mutations in the Piezo1 gene, which is integral to the maintenance of red blood cell volume homeostasis (7). Furthermore, the dysfunction of Piezo1 in adult lymphatics culminates in parenchymal lymphatic valve degeneration (8). Additionally, diminished mRNA levels of Piezo1 have been implicated in the constriction of the outflow tract and aortic valve malformation (9) among other sequelae (10).

*Piezo*1/2 channels are crucial for pain perception and initiation. They play a key role in converting mechanical stimuli into neuronal signals that contribute to pain sensation. These channels are expressed in different types of sensory neurons, including those that respond to mechanical stimuli and can be activated by physical pressure or touch (11). Activation of these channels in sensory neurons leads to depolarization of the membrane potential and opening of voltage-gated ion channels, ultimately leading to the activation of action potential firing and the perception of pain (12).

The purpose of this review was to provide a comprehensive overview of the potential association between *Piezo*1/2 and pain, as well as their pharmacological properties in managing pain.

## 2 Structure and abnormal expression of *Piezo*1/2

Patapoutian et al. initially identified the *Piezo* family of proteins in Neuro2A cells and reported that it is present in numerous species. These proteins are expressed in various vertebrate tissues including the lungs, bladder, colon, bone, sensory neurons, and DRG neurons (13). *Piezo*1/2 is a well-conserved ion channel comprising 2,500 amino acids. Various cryo-electron microscopy studies revealed that *Piezo*1 has a homogeneous trimeric structure with three blades arranged in a propeller-like configuration. It also contains a central cap structure, three long beams connecting the cap and blade structures, and a TM region (14–18). *Piezo*2 is structurally similar to *Piezo*1 but contains additional contraction sites (13) that can function as transmembrane gateways regulated by the cap domain (18).

Several studies have demonstrated that mutations in *Piezo*1 can lead to various diseases. Dehydration in hereditary stem cell disorder and congenital lymphoid dysplasia are associated with such mutations (19). Moreover, mutations in *Piezo*2 can lead to the development of distal joint contracture type 5 (DA5), as well as other diseases such as Gordon syndrome (GS) and Marden-Walker syndrome (MWS) in humans. Knocking out *Piezo*1/2 leads to embryo damage and results in the death of mice shortly after birth (20). *Piezo*1/2 is also involved in the development of diseases under corresponding pathological conditions. For example, under high venous pressure conditions, *Piezo*1 ion channel proteins can disrupt pulmonary endothelial barrier function and promote arterial remodeling during hypertension. Additionally, it is associated with aortic stenosis and the development of atherosclerosis (21). In the gastrointestinal epithelium, *Piezo*1 channels function as pressure sensors that regulate cell crowding (21) and migration- (22, 23). Wang et al. investigated the role of *Piezo*1 in large omental metastasis of gastric cancer and its underlying mechanism. *Piezo*1 expression was notably higher in 110 metastatic gastric cancer tissue samples than in non-metastatic gastric cancer tissue samples. Silencing *Piezo*1 resulted in a significant reduction in gastric cancer metastasis, whereas overexpression of *Piezo*1 inhibited apoptosis and promoted cell proliferation in GC cells. Thus, *Piezo*1 plays a crucial role in the invasion and development of gastric cancer (24). Moreover, *Piezo*1 ion channels are linked to acute pancreatitis (AP)- (23). The pancreas has a low threshold for mechanical stimulation (25). According to Romac et al. (26), injection of the *Piezo*1 agonist Yoda1 reduced the secretion of pancreatic enzymes that cause pancreatitis, and blocking *Piezo*1 prevented pancreatitis caused by mechanical stimulation. Additionally, *Piezo*1 antagonists are effective in preventing pancreatitis.*Piezo*1 promotes early local invasion of pancreatic ductal adenocarcinoma cells through a combination of environmental pH, mechanical output of pancreatic stellate cells, and stromal mechanical stimulation (27–29).

## 3 Pain behavior evaluation methods

Pain exerts a profound influence on both the physiological and psychological aspects of an individual. Behavioral alterations in patients serve as indirect indicators of the intensity of experienced pain (30). To quantify the severity of pain, a plethora of clinical assessment scales have been devised, predominantly categorized into unidimensional and multidimensional scales. Unidimensional scales, while straightforward and user-friendly, are characterized by a stronger emphasis on a singular aspect of pain assessment, which may limit their comprehensiveness. In contrast, multidimensional scales adopt a holistic approach, integrating observations, physiological responses, and behavioral changes, along with other subjective and objective indicators. This comprehensive methodology has garnered significant attention from the scientific community in recent years, highlighting the complexity and multifaceted nature of pain assessment (31).

### 3.1 Unidimensional pain assessment tool

#### 3.1.1 PAULA pain scale

The PAULA pain scale, introduced by Machata et al. in 2009, is a unidimensional tool designed for post-operative pain assessment (32). The scale comprises two sections: the upper section features a spectrum of five faces, transitioning from yellow to red, symbolizing increasing pain intensity. The lower section consists of a 20 cm long 100-grid ruler, linked to a movable scale. The scale ranges from 0 points, represented by a yellow face indicating “no pain,” to 100 points, depicted by a red face for “the most severe pain.” The PAULA scale is user-friendly, offering a seamless integration of the visual analog scale (VAS) and the facial rating scale (FRS). The initial study demonstrated that even first-time users of the PAULA scale exhibited less variability in their pain assessments compared to the VAS. In a retest involving only 23% of patients, the discrepancy in pain scores was also smaller than that observed with the VAS.

#### 3.1.2 Memorized pain assessment card (MPAC)

MPAC was initially proposed by Fishman in 1987 as a tool for the rapid assessment of cancer pain (33). The MPAC incorporates elements of the Visual Analog Scale (VAS) format, featuring three 10-cm horizontal lines to represent the intensity of pain, the degree of pain relief, and the emotional impact of pain. Each line is anchored by endpoints labeled “least” and “most.” Additionally, the second part of the card utilizes a modified Tursky pain descriptor scale. Fishman examined the correlation of these four components with the Zung Anxiety Inventory, Hamilton Depression Scale, and MPO, confirming their consistency (34). The MPAC's reliability and validity have been established. While the MPAC integrates various dimensions of pain assessment, it is categorized as unidimensional due to its focus on pain intensity as the primary measure, akin to the PAULA Pain Scale. This integration enhances the comprehensiveness of pain assessment while maintaining a unidimensional approach.

#### 3.1.3 Critical-pain observation tool (CPOT)

The CPOT was developed by Gelinas et al. in 2006, specifically for assessing pain in critically ill patients who may be unable to communicate their pain levels (35). The CPOT is characterized as a unidimensional scale focusing on behavioral indicators of pain. It comprises four behavioral items: facial expression, body movement, muscle tension, and compliance with ventilation (for intubated patients) or vocalization (for non-intubated patients). Each item is scored from 0 to 2 based on the patient's observed responses, resulting in a total score ranging from 0 to 8. A higher total score indicates a greater intensity of perceived pain. Although the CPOT assesses multiple behavioral aspects, it is considered unidimensional because it aggregates these observations into a single score representing the overall pain intensity.

### 3.2 Multidimensional pain assessment tool

There are three common pain assessment tools: the short-form McGill Pain Questionnaire (SF-MPQ) (36), the Brief Pain Inventory (BPI) (37), and the Global Pain Scale (GPS) (38). The SF-MPQ, developed by Melzack, includes a pain rating index, a visual analogy rating method, and existing pain intensity. It employs a pain rating scale with 15 descriptive pain words to reflect the nature of pain, and quantitatively describe the degree of pain. The BPI, however, is a quick multidimensional pain assessment scale with 15 entries for pain and pain levels (39). It has been translated into several versions, including a Chinese version, and is used to assess neuropathic pain in patients with urolithiasis and cancer. Another tool, the GPS, developed by Gentile et al., includes four dimensions: pain, emotion, clinical manifestations, and daily activities. Scores range from 0 to 10 for each item and can reach a maximum of 200, reflecting pain severity. It is primarily used to access chronic pain and can provide valuable assessment and treatment plans for healthcare professionals (40).

## 4 *Piezo*1/2 and cancer pain

Pain associated with malignant tumors, including epithelial cancers and mesenchymal sarcomas, has become one of the most widespread health concerns worldwide (41). Tumors often occur as a result of clonal abnormalities in the level of gene regulation, which manifest themselves as developmental dedifferentiation, structural heterogeneity, persistent growth, and organismal incoordination (42–44). In recent years, evidence showing a high correlation between *piezo* genes and tumorigenesis has increased in a stepwise manner. Further, *piezo* ion channels are aberrantly expressed in a variety of cancers, including but not limited to respiratory cancers (45), the expression levels of piezoelectric proteins in different types of tumors and the related mechanisms are shown in Table 1, genitourinary cancers (46, 47), and gastrointestinal cancers (22), disrupting pre-existing normal electrical-mechanical signaling and participating in the development of pain in cancer (48).

**Table 1:** Expression levels and related mechanisms of *piezo* in various types of cancer.

Cancer types	*Piezo* channel	Research model	Expression	Role in cancer	Prognosis	Possible mechanisms	References
Prostatic cancer	Piezo1/2	DU145 and PC3 PCa cell lines	Upregulation	Migration, Proliferation	n/a	Involved in Akt/mTOR signal transduction	(48)
Breast cancer	Piezo1/2	Malignant MCF-7 breast cancer cell line, MCF10A cells and MDA-MD-231 cells	Upregulation	Migration, Proliferation, Invasion	Poor	Activation of the Akt signaling pathway	(50, 51)
Bladder cancer	Piezo1/2	Bladder tissue of Balb/c strain mice	Upregulation	Migration, Proliferation, Invasion	Poor	Unknown	(52)
Gastric cancer	Piezo1	Gastric cancer cell lines	Upregulation	Tumorigenesis, Migration, Invasion	Poor	As TFF1 binding protein	(23, 24)
Colorectal cancer	Piezo1	Colon cancer tissue	Upregulation	Proliferation, Migration	Poor	Interference with the *Piezo*1-Mcu-HIF-la-VEGF signaling pathway	(53)
Oral squamous cell Carcinoma	Piezo1	U373-MG and U87-MG human glioblastoma cells	Upregulation	Proliferation, Migration, Invasion	n/a	YAP signal	(54, 55)
Lung cancer	Piezo1/2	Human non-small cell LC(NSCLC) tissue	Downregulated	Tumorigenesis, Migration, Invasion	Good	Unknown	(46, 56)
Glioma	Piezo1/2	Glioblastoma tumor tissue	Upregulation	Angiogenesis, Apoptosis resistance, Proliferation	n/a	Mechanical transduction, vascularization	(57, 58)
Osteosarcoma	Piezo1	Human osteosarcoma (OS) cells	Upregulated	Oncosuppressor	n/a	Abnormal mechanical transduction	(59, 60)

n/a, not available.

### 4.1 Relationship between *piezo* channels and genitourinary tumors

Prostate cancer is a malignant tumor that predominantly affects men and is accompanied by painful mechanical compression in addition to invasion of the glandular tissue itself (49). The generation of pain is closely related to the pressure on the internal organs, infiltration of cancer cells, and expansion of cancerous tissue, which is highly diffuse. Immunohistochemical analysis revealed that the *piezo*1 gene is significantly higher in prostate cancer cells than in normal prostate and paracancerous tissues (47). Yu et al. compared TCGA and GENT1 databases and found that *piezo*1 expression levels in cancer tissues and normal tissues were not the same; in the TCGA database *piezo*1 was more expressed in normal tissues, whereas in the GENT1 database, the opposite was true (50). Elevated piezo1 activity is also observed in the cancerous tissues of bladder cancer patients, and excessively open **mechanosensitive ion channels** (MSC) contributes positively to the metastatic proliferation of cancer cells; however, this adversely affects the body's battle against cancer and ongoing survival (51, 52).

### 4.2 Relationship between *piezo* channels and digestive system tumors

Cancers of the digestive system, including those of the stomach (53), intestines (54), and upper gastrointestinal tract (55), are the most common malignancies. There are many causes of pain, including but not limited to compression pain and obstructive pain caused by rapid tumor growth, as well as pain caused by tumor infiltration of blood vessels, resulting in arterial occlusion, venous stasis, and local hypoxia (56, 57). In normal human gastric GES-1 cells, *piezo*1 can act as a trefoil factor family 1 (TFF1) binding protein that mediates cell proliferation and migration, whereas in gastric cancer cells, with knockdown of the *piezo*1 channel, the cell cycle is stalled in the G0/G1 phase and cell proliferation and migration are reduced (22, 23). Furthermore, if *piezo*1 extracted from gastric cancer cells is injected into mice, tumor growth can be inhibited (58). Colorectal and upper gastrointestinal tract cancers, such as oral squamous epithelial cancer, are two additional gastrointestinal cancers linked to *piezo*. The upregulation of *piezo*1 expression plays a role in both their development and progression, but the two act on slightly different signaling pathways, which achieve further tumor advancement by interfering with the *Piezo*1-Mcu-HIF- la-VEGF signaling pathway in colorectal cancer, and are associated with YAP signaling in oral epithelial cancer (59–61).

### 4.3 Relationship between *piezo* channels and lung cancer

In contrast to the aforementioned cancers, the development of lung cancer is associated with a significant downregulation of Piezo1 and Piezo2 gene expression. Specifically, in non-small cell lung cancer (NSCLC), both Piezo1 and Piezo2 mRNA translation levels are notably reduced compared to adjacent lung non-tumor cells. This downregulation indicates a high degree of deletion in gene expression within NSCLC. Furthermore, when Piezo1 and Piezo2 are experimentally knocked down, lung cancer tissue primary foci exhibit an increased growth trend and a significant enhancement of *in vitro* metastasis (45). Moreover, this metastatic trend and pain sensation were closely related to the degree of piezo gene deletion. In the early stage of lung cancer, that is, when piezo gene expression is initially lost, patients do not have pain sensation or feel mild pain, but as the cancer progresses, the pain sensation can progressively deepen and metastasize, causing diffuse pain in the chest wall, which can be accompanied by pain radiating to the shoulder and other places (45, 62). Despite the lack of understanding of the underlying mechanism, the use of appropriately increased *piezo* gene expression as a major direction for lung cancer treatment is promising.

### 4.4 Relationship between *piezo* channels and other cancers

Gliomas are neurologically related tumors that cause persistent and severe headaches and originate from glial cells in the brain, and *piezo*2 is involved in the regulation of the vascular architecture of their tissues (63, 64). This role is associated with calcium ions, and the inward flow of calcium ions enhances Wnt signaling activity, enabling serial signaling of Wnt and β-proteins and promoting vascular activity and permeability in glial cells (63). In a *Drosophila* model analysis, *piezo*1 could accelerate glioma development by enhancing the mechanical transduction of tissue (65). The pain caused by glioma is neuropathic cancer pain, which differs from the injurious pain caused by several of the aforementioned cancers in that, in addition to the onset of hypersensitivity symptoms (persistent tingling sensation as well as a burning sensation), it is accompanied by hypoallergenic symptoms (numbness and decreased electrical conduction ability) (66, 67). Osteosarcoma (OS) is the most common malignant bone tumor in adolescents. As an aggressive class of aberrations, their occurrence is closely associated with aberrant mechanotransduction, often accompanied by overexpression of *piezo*1 in OS cell lines, a feature also observed in the SW982 synovial sarcoma (68, 70). In addition to the pain caused by OS itself, bone pain is one of the most common types of pain in cancer patients, and its onset is often spontaneous and progressively worsens as the disease develops, progressing from an initial intermittent dull ache to unbearable pain (71–73). This pain often manifests as **a breakthrough pain**, that is, recurrent episodes of extreme pain (72).

Similarly, *piezo*1 showed differences in mRNA expression levels, with its expression being significantly higher in the PC3 and DU145 cell lines than in the normal cell line RWPE-1 (47). This implies that increased expression of the piezo1 gene contributes to prostate cancer progression, as evidenced by the invasive proliferation of cancer cells and escalated visceral pain. In the development of breast cancer, overexpression of *piezo*2 contributes to the low survival rate of triple-negative breast cancer (TNBC), suggesting that the opening of excessive *piezo*2 channels enhances the proliferative capacity and invasiveness of TNBC cells, which may be related to the activation of the Akt signaling pathway (46). The opening of gated *piezo*2 channels allows the release of calcium ions from the sarcoplasmic reticulum, accompanied by a Ca2+ inward flow, triggering the RhoA-mDia pathway, which in turn regulates the actin-constituted cytoskeleton (74, 75). Notably, *piezo*1 is also involved in breast cancer-related processes and plays different roles in changing breast cancer type and stage.

## 5 *Piezo*1/2 and inflammatory pain

**Figure 1:**
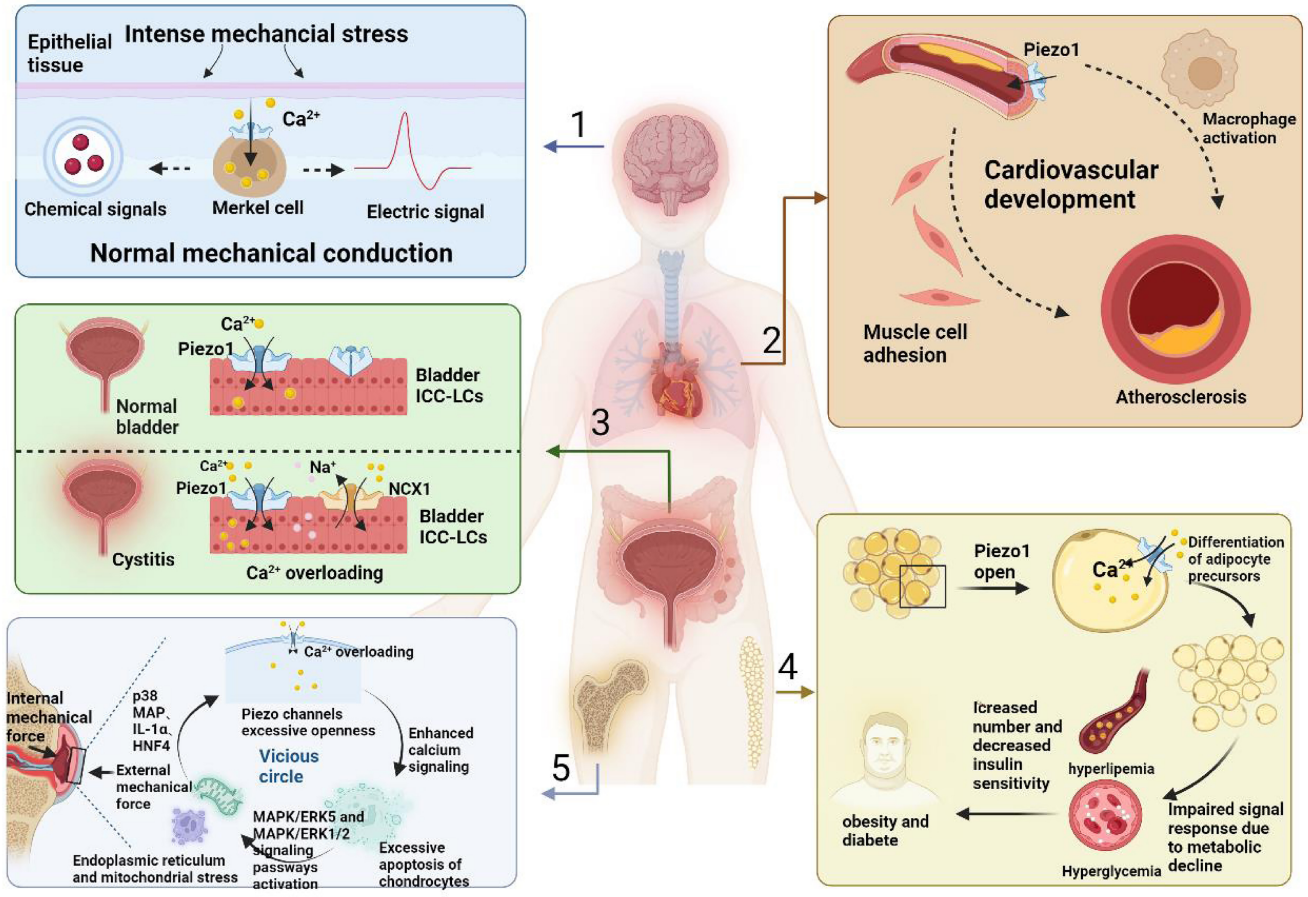
The expression of piezo1/2 in various inflammatory diseases throughout the body.

Stimulation by mechanical forces occurs throughout human life, and the source of this stimulation can be either passively applied or generated intracellularly (76, 77). When the stimulation increases beyond the maximum stimulation intensity that the tissue cells can withstand, the release of cytokines such as *TNF-*α, *IL-1*β, *IL-6,* and *IL-8* can be observed, inducing inflammation (78–80). Cells can produce corresponding responses through the mechanical transduction of harmful stimuli (13). Inflammation can be divided into acute inflammation, which is dominated by metaplasia and exudation with visible infiltration of neutrophils (21, 81), and chronic inflammation, which is a class of proliferative reactions characterized by the infiltration of lymphocytes and plasma cells (82). Acute inflammation is essentially a defense response in living tissues, with the vascular system being stimulated by external and internal damage factors. This represents not only the predominant pathological phenomenon in humans and numerous animals, but also the key defensive mechanism that, significantly contributes to the defense against pathogenic incursion, infection, and mending of cell tissues (83). In contrast, chronic inflammation, which usually lasts six weeks or longer, brings about unpleasant subjective and emotional experiences associated with tissue damage or potential damage (84). During the transition from acute to chronic inflammation, cytokines mediate the migration of immune cells and their adhesion to cells at the site of injury, leading to the infiltration of inflammation-associated cells. Inflammatory cells, while removing necrotic cells, also cause damage to the surrounding normal cells, promoting the release of growth factors by the organism, stimulating the proliferation of new cells with structural morphology and physiological function different from normal cells, and transforming inflammation into chronic inflammation (85). The expression of piezoelectric protein 1/2 in various systemic inflammatory diseases is shown in Figure 1.

*Piezo*1/2 channels are a class of MSC capable of converting mechanical signals into electrochemical signals; *piezo*1 plays a role in pain that is still under investigation, while *piezo*2 has been shown to play a role in many types of pain (86, 87). For example, *piezo*1 is essential for controlling vascular blood pressure, mainly by mediating the release of ATP and participating in *P2Y_2_/G_q_/G_11_*-mediated activation of downstream transduction signals, which leads to the phosphorylation and activation of protein kinase B(AKT) and endothelial NOS. In contrast, in mice with *piezo*1 *KO*, we observed a loss of vasodilatory capacity and an inability to form *NO*, manifesting as hypertension (88). Atherosclerosis (AS) is a multifactorial chronic inflammatory disease caused by damage to the vascular endothelial cells (ECs) and is essentially a class of chronic inflammatory diseases (89). During atherosclerosis development, *piezo*1 is involved in EC injury and mediates macrophage activation. At the same time, ECs can attract and adhere to muscle cells because of their stimulatory effect (21, 89). Inflammatory reactions in the bone and joints are a worldwide health problem, especially in weight-bearing joints, resulting in reduced mobility and significant medical costs (90). The researchers found that interleukin-1α (IL-1α) effectively upregulated *piezo*1 expression in chondrocytes and induced an increase in Ca^2+^ concentration in the intracellular fluid, accompanied by the opening of *piezo*1/2 ion channels during chondrocyte perception of inflammation and mechanical trauma (91). This results in a dilution of F-actin and greater susceptibility to mechanical trauma. In the development of osteoarthritis (OA), *IL-1*α does not act in isolation, but also in concert with several protein kinases and transcription factors, such as *p38 MAP*, *HNF4,* and *ATF2*/*CREBP1 (*91, 92).

Gentle mechanical stimulation, such as touch, does not cause pain but is accompanied by pain after tissue damage and inflammation occur (93). In contrast, when *piezo*2 KO mice and *piezo*2-deficient humans were similarly stimulated by touch, no signs of overactivity were observed (18). Recently, it has been shown that the knockdown of *piezo*2 channels can completely disable neurons that cause mechanical pain in mice (94) and that this removal not only results in reduced sensitivity to stroking stimuli but also an increase in the pain threshold (95, 96). This suggests that *piezo*2 is essential for mechanical and inflammatory pain because the body's response to small stimuli such as touch and vibration requires *piezo*2 involvement (94). One theory is that in many diseases of unknown origin, such as amyotrophic lateral sclerosis (ALS) and dry eye disease, piezo2 is involved in a signaling system that malfunctions. The changes in piezo2 channel, which is involved in transmitting proprioceptive signals, interrupt the transmission of consciousness proprioceptive signals to the hippocampus and disrupt the feedback process of motor neurons’ ultrafast proprioceptive signals (97). piezo2 has also been linked to acute pain. Studies on mice have shown that piezo2 is essential for the sensitivity of the mechanical stimuli that can cause acute pain, namely, that piezo2 depends on the nociception that is necessary for the mice to experience acute pain (98).

In addition to the typical inflammatory diseases described above, *piezo1/2* channels can be expressed to some extent in various inflammatory conditions, such as fibrosis in the lung (a group of lesions characterized by an inflammatory response, fibroblast proliferation, lung tissue damage, and extracellular matrix deposition) (99), obesity and diabetes (hypertrophy and hyperplasia of adipocytes, which in turn cause inflammatory effects in adipose tissue with insulin resistance) (100) and chronic cystitis (101), which brings about painful reactions of varying degrees.

The numbered tissues are as follows: (1) CNS, (2) cardiovascular system, (3) genitourinary system, (4) adipose tissue, (5) bone tissue. When the organism receives external inflammatory stimuli, it first transmits them through the piezo1/2 channels and transforms them into electrical and chemical signals, which subsequently cause histological changes with different morphologies in different tissues. In the cardiovascular system, monocyte activation and myofibroblast adhesion are induced, accelerating the progression of atherosclerosis, in the genitourinary system there is an overload of calcium inward flow and dysfunction of ICC-LC receptors, in the adipose tissue there is a decrease in the hypertrophy and maturation of progenitors, and in the chondrocytes, there is inflammatory effusion, further damaging the newborn osteoblasts.

## 6 *Piezo*1/2 and neuropathic pain

The central nervous system (CNS), upon receiving mechanical signals from its environment, is capable of exhibiting diverse alterations, such as growth and diversification, movement and bonding, and structural modifications, encompassing nociceptive transmission (102–104). Neuropathic pain is a kind of pain caused by abnormal somatosensory system and disturbance of sensory signal transmission to the spinal cord and brain. Neuropathic pain is usually a chronic and secondary pain that can predict abnormalities in the peripheral nervous system or central nervous system (66). Approximately 500 million people worldwide suffer from it (105). For *piezo*1, Koser DE et al. showed that its presence is observed in retinal ganglion cells (RGCs) of African clawed toads (106, 107). RGCs are an important component of the CNS (108). RGC axons respond to mechanical signals transmitted *in vivo*, and when RGC embryos were treated with the *piezo* channel inhibitor GsMTx4, or when gene expression was downregulated by approximately 42% using morpholino knockdown, an irregular trend of axon spreading was observed, accompanied by a reduction in length and a decrease in the ability to transmit signals (107). *Piezo*2 is a class of genes homologous to *piezo*1 that, when aberrantly expressed, can render cells with low mechanical stimulation thresholds hypersensitive (109), and whose expression is more selective and enriched in somatosensory neurons (12).

### 6.1 *Piezo* channels and central neuropathic pain

**Figure 2:**
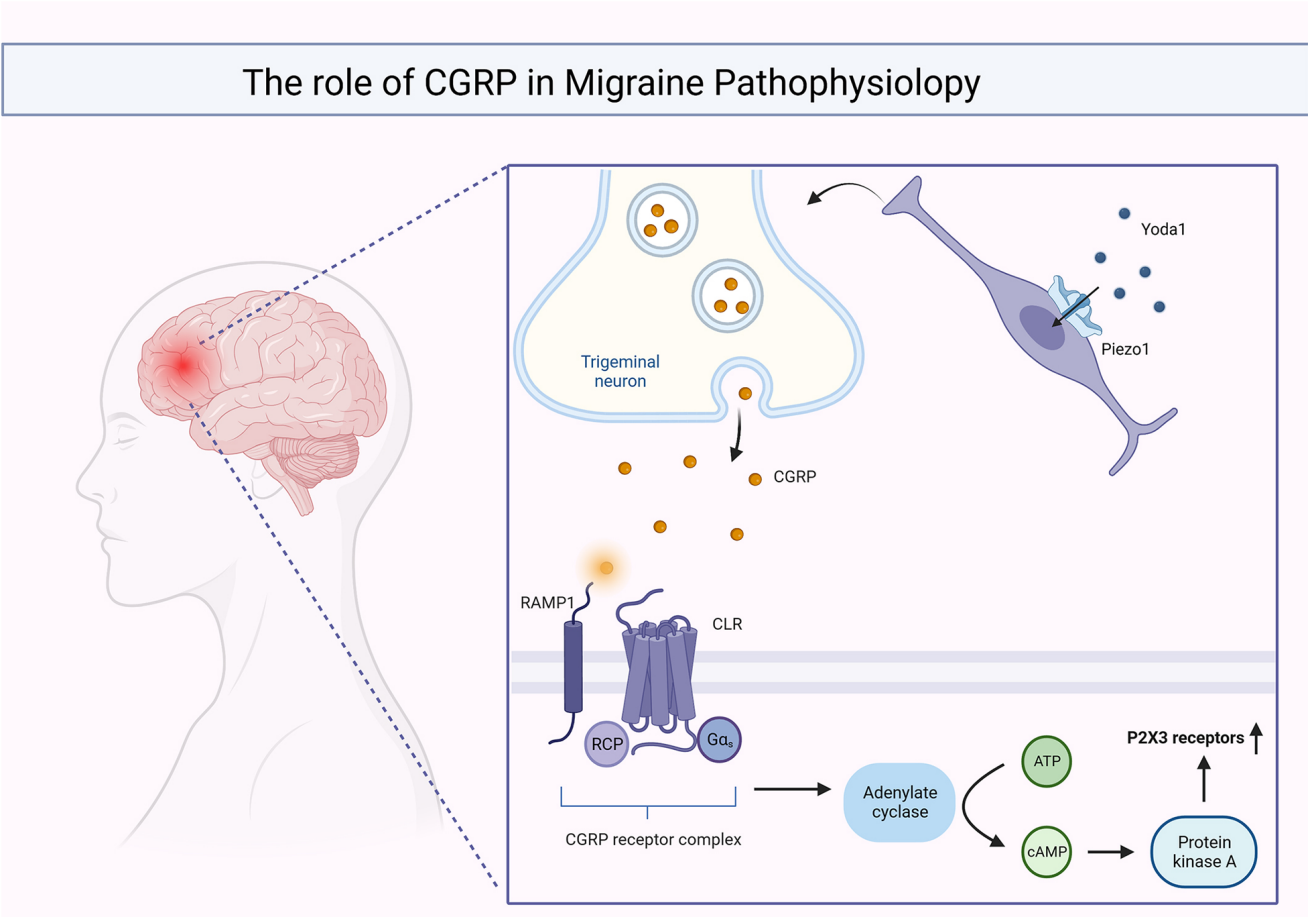
CGRP and *piezo* channels are involved in migraine onset and function at different stages.

According to this classification, neuropathic pain can be further classified into central and peripheral nerve pain (110). The first type of pain is often caused by lesions in the spinal cord or brain. Migraine is the most common type of representative. Migraine is a common chronic neurovascular disease and one of the most serious disabling diseases in the world (111). As a group of neurological disorders characterized by persistent headaches, it originates from the vascular injury receptor system located within the trigeminal nerve and is closely associated with hypersensitivity reactions (112). Adriana Della Pietra and team found that in the sensory neurons of the trigeminal nerve (113), the development and closure of both *piezo*1 and *piezo*2 channels can be detected, which are involved in the control of facial muscles and, micro-expressions while being able to induce the production of pulsatile neuropathic pain, one of the qualities of migraine (114). The opening of the *Piezo*1 channel can be achieved using exogenous facilitators. Yoda1 is a class of small lipid-soluble substances that is specific for *piezo*1 and which cannot act on *piezo*2 (115). When Yoda1 binds to one of the three subunits of the *piezo*1 channel protein, it interacts with the carboxyl end of the peptide chain of the *piezo*2063 protein to open the channel (116, 117). Calcitonin Gene-Related Peptide (CGRP) is the main mediator of trigeminal nerve sensitization and its action depends on the activation of protein kinase A (PKA) and PKC (118). CGRP binds to its corresponding receptors and activates adenylate cyclase through G proteins, with the assistance of cAMP to activate PKA and PKC to achieve signal amplification and ultimately enhance the expression of *P2X3* receptors, P2X3 receptors sensitizes sensory nerve fibers, increasing their responsiveness to pain signals and leading to allodynia (119–121). In summary, the onset and development of migraine are closely related to the activation of meningeal injury receptors (122), which are mechanosensitive and accompanied by the involvement of *piezo* channels (123). CGRP and piezoelectric channels are involved in the pathogenesis and different stages of migraine as shown in Figure 2.

### 6.2 *Piezo* channels and peripheral neuropathic pain

The other category is peripheral neuropathic pain, often caused by damage to peripheral conduction nerve fibers, especially Aα and Aβ fibers (124). After peripheral nerve injury, the anterior cingulate cortex (ACC) exhibits excessive excitability accompanied by the upregulation of *piezo*1 gene expression, which is involved in pain processing (125). Qiao-Yun Li et al. showed that, using a mouse sciatic nerve peripheral nerve injury model, it was able to effectively stimulate the intermediate neurons, increase the opening of *piezo*1 channels in the bilateral ACC, mediate the inward flow of extracellular Ca^2+^, release Ca^2+^ from the sarcoplasmic reticulum, and finally induce phagocytosis of microglia (125). Up to now, there are relatively few reports on the potential role of piezoelectric 1/2 in other neural injury models, such as chemotherapy induced peripheral neuropathy, spare nerve injury, sciatic nerve ligation, and even tumor bone metastasis. Recent studies have shown that mice lacking Piezo2 in their tail sensory neurons exhibit impaired nociceptive responses to mechanical stimuli, and suggest that targeting Piezo2 may be an effective strategy for treating mechanical allodynia (94). However, of note, excessive *piezo*1 expression often signals an influx of excess Ca^2+^ (126, 127), which may result in the emergence of oxidation or the weakening of the axonal skeleton during the process of repairing nerve damage- (128, 129), inducing neurodegeneration, which is often irreversible (130).

When Yoda1 acts on *piezo*1, it results in the release of large amounts of intra-synaptic CGRP, which binds to the CGRP receptor complex and activates the cAMP/PKA signaling pathway, ultimately leading to an increase in *P2X3* receptor expression.

## 7 The related antagonists and pharmacological properties of *Piezo1/2*

Various types of pain are present in all aspects of life, not only causing substantial damage to the body but also being psychologically accompanied by great stress (131–133). The occurrence of cancerous pain predicts local tumor proliferation in the organism (42, 133). Inflammatory pain is accompanied by mechanical stimulation of various types of silver injury (83, 90) and neuropathic pain is closely related to the destruction of central or peripheral nerve fibers (134). Despite the strong self-healing capacity of the organism, it is somewhat helpless when confronted with these types of unfavorable pain, because they do not have a specific pathogen, such as a bacterial infection or viral invasion (135–137). Therefore it is necessary to conduct drug research on the implicated illnesses.

The involvement of *piezo* channels in mechanical signaling between various cells or tissues has been confirmed by animal experiments (138), such as the establishment of Drosophila (139, 111), birds (140), and mice (141, 142). As already mentioned, pain, whether physiological or pathological, is often accompanied by an abnormally large opening of *piezo*1/2 channels. Therefore, the use of efficient antagonists and blockers to inhibit the expression of *piezo* genes or the opening of channels will help treat related diseases (51, 90, 143). The details of this process are described below. A variety of piezoelectric channel antagonists and their related properties are shown in Table 2.

**Table 2:** Various antagonists of piezo channels and their related properties.

Antagonist name	Selectivity	Drug properties	Acting sites	Mechanism of action	*in vivo* evidence for Piezo channels	References
RR	Nonselective	A polycation	Extra-membrane calcium channels	Generic voltage-sensitive inhibitors of calcium channels	*in vivo* injection in mice and Drosophila	(115, 149)
Gd^3+^	Nonselective	Lanthanide trivalent metals	Ion channel pore	Universal Inhibitors	None	(167, 168)
GsMTx4	Nonselective	Peptide toxin	Ion channel pore	Regulation of local membrane tension near the channel	Intracorporeal injection of mice	(161, 169, 170)
Tubeimoside1	Selective	Traditional Chinese Medicine	Yoda1 binding site	Tubeimoside1 competitive inhibition	None	(94, 165)
Dooku1	Selective	Compound	Yoda1 binding site	Combined with the Yoda1 pyrazine ring	None	(164, 168)

### 7.1 Antagonistic effects of non-specific drugs

*piezo*1/2 channels are a complex, novel, and unique class of MSCs, for which pharmacological studies are still in their infancy, and the antagonists identified so far lack specificity and cannot target *piezo* channels efficiently. Ruthenium red reagents (RR), gadolinium metal (Gd^3+^), and GsMTx4 are the most commonly used *piezo*1 and *piezo*2 blockers (144, 145). RR is a polycation (146). In previous studies, experimentalists did not explore the mechanism of action of RR clearly but only pointed out that the concentration of the reagents might be an influencing factor, and the opening of *piezo* channels showed a decreasing trend with increasing RR concentration (147). However, recent studies have shown that RR treatment of cells outside the cell membrane only blocks *piezo*1/2 channel-mediated inward currents but has no effect on outward currents, suggesting that our RR action may be based on an extra-membrane blocking mechanism (148). The lanthanide trivalent metal gadolinium is often found in various experiments on mesenchymal cell growth and development (51). which can inhibit not only TREK-1(A potassium channel that controls cell excitability and keeps the membrane potential below the depolarization threshold is involved in neuropathic pain) pathway and voltage-gated sodium channel, but also piezoelectric channel (149, 150).

Compared to RR and Gd^3+^, GsMTx4 is more widely used in MSC. This amphoteric polypeptide, abundant in cysteine (six), initially derived from the tarantula venom, is currently one of the blockers of MSC (151–153). GsMTx4 is an inhibitory cysteine linking (ICK) peptide, ICK peptides tend to have both hydrophobic and hydrophilic sides, and thought to act by inserting their hydrophobic side into the cell membrane and binding to the ion channel voltage sensor domain on their hydrophilic side (17, 25). This amphiphilic nature effectively facilitates their attachment to the lipid bilayer, enables the binding of membrane-gating elements, mediates the transduction of signaling molecules, and ultimately alters the kinetic effects on the cell membrane (154, 155). The ICK peptide GsMTx4 isn't the sole variant extracted from tarantula venom; it is merely a standard example that shows minor variations from other ICK peptide types (155, 156). Most ICK peptides act stereoisomerically, which is not the case for GsMTx4 (157, 158). We found that both L-GsMTx4 and D-GsMTx4 exerted almost equal inhibitory effects, and it is reasonable to assume that this is due to the high positive charge (+5) and hydrophobicity of GsMTx4 and the high proportion of lysine content (159, 160). While the previously described inhibitor RR exerts its effect by blocking action by binding to gating elements on biological membranes (148), GsMTx4 takes a very different approach in which its proximity to the *piezo* channel is not selectively inserted into the phospholipid bilayer (161), achieving an increase in tension on both sides of the membrane and a decrease in lateral pressure (147, 162), which ultimately induces relaxation of the outer membrane and decreases the efficiency of force transduction by mechanical stimulation (144).

Pain generation coincides with the activation of ion channel gates, indicating that there is a disproportion of cations, particularly calcium ions, with non-selective cationic MSC playing a key role in this occurrence.D-GsMTx4 is an effective protective agent in ischemia-reperfusion (causing myocardial pain with infarction) that occurs in the myocardium (163). In experiments exploring the relationship between *piezo*1 channels and the inflammatory response, it was found that the blockade of MSC channel opening with GsMTx4 was accompanied by a corresponding increase in certain cytokines that contributed to the reduction of inflammatory pain in the CNS, the more prominent ones being interleukins (such as IL-1β, IL-6, and IL-8) and tumor necrosis factor (164).

### 7.2 Potential specific blocking agents

Notably, these three inhibitors lack specificity; however, recent research indicates Tubeimoside1 and Dooku1 may specifically target *piezo* channels (164). Mechanical signaling is not the only factor that can activate *piezo* channels; the small molecules Yoda1 and Jedi2/1 can also selectively activate *piezo*1 channels (117). During the research conducted by Evans et al., the influence of Tubeimoside1 lies not in altering piezo1 channels’ function, but in its reliance on Yoda1, meaning the impact of Tubeimoside1 is based on Yoda1's existence, a dependency also observed in Dooku (165, 166). The effect of Tubeimoside1 decreased gradually with the increase of Yoda1 concentration and the inhibition was reversible, suggesting that Tubeimoside1 competes with Yoda1 to bind *piezo*1 channel, and when the same experiment was conducted on TRPV4, TRPM1, and TRPC5 belonging to MSC, no significant inhibition of Tubeimoside1 was found, which again proved that it might be a specific antagonist of the *piezo* channel (166, 167). Yoda1, a chemically synthesized small molecule, has a pyrazine ring within its structure that serves as the exact binding site for Dooku1. While Dooku1's sole activation of constitutive *piezo*1 channels does not lead to inhibition, it suppresses native Yoda1 and reduces the activation of associated channels (165).

## 8 Conclusion

Pain frequently occurs in numerous clinical scenarios, profoundly impacting a patient's quality of life. Consequently, researchers have focused extensively on employing efficient techniques to alleviate pain and distress in patients. Owing to the complex pathological mechanisms of pain, drugs are commonly used to relieve pain clinically; however, their therapeutic effects are poor. The role of *Piezo*1/2 in pain is widely accepted. In this review, we discuss the pathogenesis of pain and its relationship between *Piezo*1/2 and pain. Consequently, creating and employing targeted *Piezo*1/2 antagonists to inhibit its excessive expression may have clinical significance in pain management.
